# Study on the Hygroscopic Properties and Mechanism of Novel Melt-Cast Matrix 3,4-Dinitropyrazole (DNP)

**DOI:** 10.3390/molecules30234644

**Published:** 2025-12-03

**Authors:** Tong Guan, Yuehui Yue, Wujiang Ying, Bo Yan, Pan Liu, Xiangrong Zhang

**Affiliations:** 1 State Key Laboratory of Explosion Science and Safety Protection, Beijing Institute of Technology, Beijing 100081, China; bitgt158100@163.com (T.G.); 3220245075@bit.edu.cn (Y.Y.); 3120215177@bit.edu.cn (W.Y.); 2 Gansu Yinguang Chemical Industry Group Co., Ltd., Baiyin 730900, China; ywj892@gmail.com; 3 Chongqing Hongyu Precision Industry Co., Ltd., Chongqing 402760, China; 18810939321@163.com

**Keywords:** 3,4-Dinitropyrazole, hygroscopicity, melt-cast explosive, critical relative humidity, hydrogen bond

## Abstract

3,4-Dinitropyrazole (DNP) is a promising candidate as a next-generation matrix for melt-cast explosives. However, its hygroscopicity severely limits the application of DNP. In this work, the macroscopic hygroscopic properties of DNP powder and charge were determined through moisture absorption tests under varying temperature and relative humidity (RH) conditions. At the micrometer scale, the morphological evolution after moisture absorption was observed by scanning electron microscopy (SEM). The moisture absorption mechanism of DNP at the molecular level was elucidated using Raman spectroscopy. The results demonstrate that the hygroscopicity of DNP intensifies with rising temperature and RH. The critical relative humidity (CRH) was determined to be 85% at 25 °C, 62% at 40 °C, and 42% at 55 °C. The surface of dried DNP particles exhibits a highly developed porous structure conducive to moisture adsorption from the environment. The moisture absorption mechanism of DNP involves water molecules forming hydrogen bonds with both the N–H bonds and nitro groups of DNP molecules. The hydrogen bonds between water and DNP molecules replace the original N-H···O/N hydrogen-bond network within the DNP crystal and disrupt the intermolecular π-π stacking interactions.

## 1. Introduction

**Figure 1:**
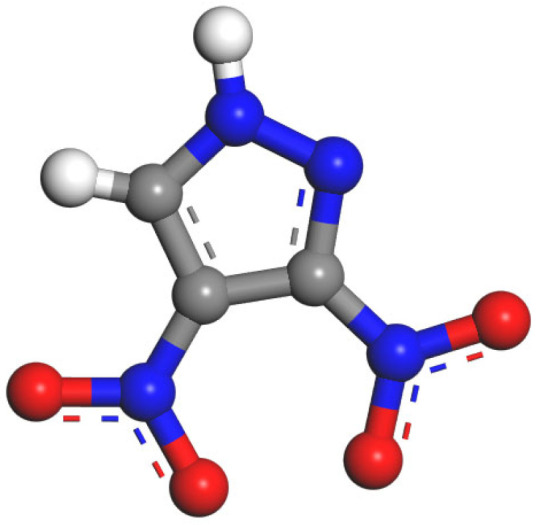
Molecule structure of DNP.

In the field of energetic materials, 3,4-dinitropyrazole (DNP, Figure 1) has attracted considerable interest owing to its exceptional detonation and safety performance [1]. DNP demonstrates higher detonation velocity, detonation pressure, and detonation heat than 3,4-dinitrotoluene (TNT), while maintaining comparable mechanical and thermal sensitivities [2,3]. This makes DNP a promising candidate as a next-generation matrix for melt-cast explosives. However, DNP exhibits hygroscopicity and acidity [4]. ($pK_{a}$ = 5.14 [5]). Moisture absorption leads to a reduction in the detonation performance and mechanical properties of the explosive charge [6,7]. Moreover, after deliquescence on the charge surface, the resulting aqueous DNP solution can corrode ammunition casings (e.g., steel and copper). Thus, investigating the moisture absorption properties and mechanisms of DNP is essential to mitigate its hygroscopicity and advance its practical applications.

Hygroscopicity describes the ability of a substance to absorb moisture from the environment under specific temperature and relative humidity (RH) conditions [8]. Hygroscopic energetic materials are not uncommon, such as ammonium dinitramide (ADN), ammonium nitrate (AN), and ammonium perchlorate (AP). The moisture absorption curve and isotherms of water vapor sorption are typically determined using the dryer balance method [9,10,11,12], yielding the critical relative humidity (CRH). Below the CRH, moisture absorption is negligible; above it, the substance continuously absorbs moisture until it ultimately deliquesces into a saturated solution [13,14]. Thus, the CRH serves as a measure of a material’s hygroscopic strength. At 25 °C, the CRH of ADN, AN, and AP are 55.2% [15], 62.7% [16], and 94.2% [17], respectively. However, the hygroscopic properties of DNP have not been reported. Interactions between substances and water molecules can be elucidated through various spectroscopic tests and molecular dynamics simulations [18,19]. Existing studies indicate that ADN, AN, and AP are all ionic crystals with hydration energies exceeding lattice energies. Their ionic groups readily interact with water molecules via electrostatic forces and hydrogen bonding [20,21,22], leading to surface dissolution. In contrast, DNP is a molecular crystal stabilized primarily by intramolecular hydrogen bonds and π-π stacking interactions [23,24]. Reference [25] has calculated possible binding conformations between DNP and water molecules using density functional theory (DFT). However, the mechanism underlying its moisture absorption remains entirely unexplored.

This work systematically investigated the hygroscopic properties and mechanisms of DNP across multiple scales. The macroscopic hygroscopic properties of DNP powder and charge were first determined through the moisture absorption curves under varying temperature and RH conditions. Progressing to the micrometer scale, the morphological evolution of DNP at different moisture contents was observed. The interaction process between DNP and water molecules was elucidated at the molecular scale through Raman spectroscopy, providing a theoretical basis for addressing the hygroscopicity of DNP.

## 2. Results and Discussion

### 2.1. Macroscopic Hygroscopic Properties of DNP Powder and Charge

**Figure 2:**
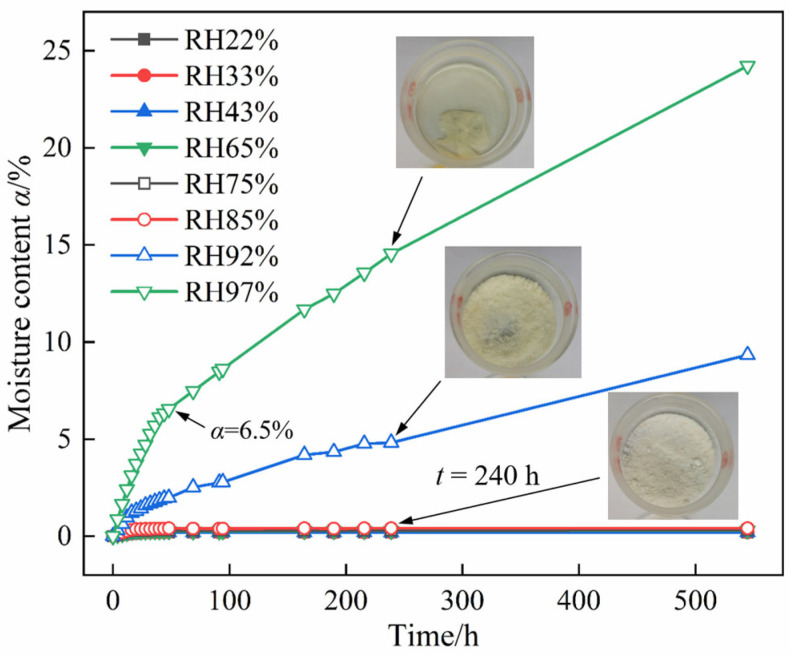
Moisture absorption curves of DNP powder at different RH values and 25 °C.

**Figure 3:**
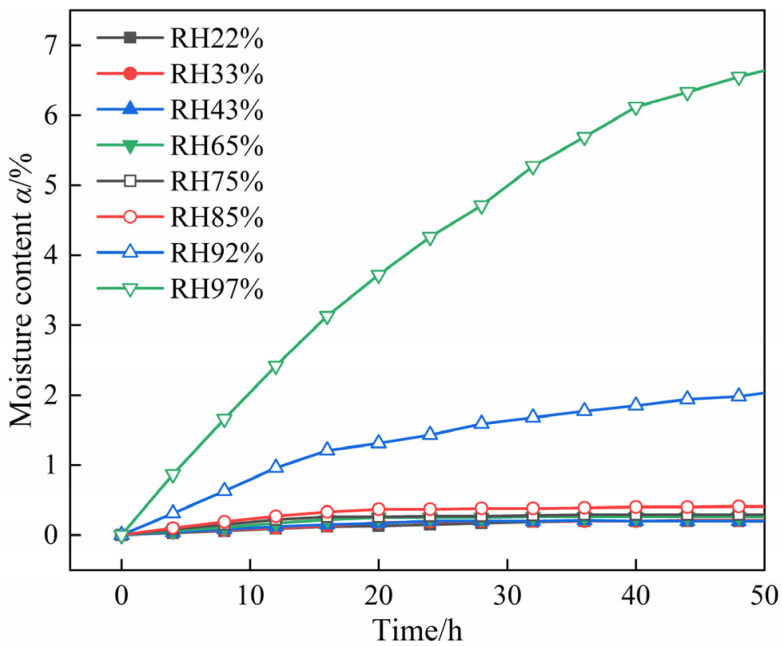
Partial enlarged view of Figure 2.

Figure 2 and Figure 3 present the moisture absorption curves of DNP powder at 25 °C under different RH conditions. At 85% RH, the sample remained a white powder without agglomeration or deliquescence after 240 h, as illustrated in Figure 2. Furthermore, at this RH level, the sample reached an equilibrium moisture content of 0.41% within 20 h (Figure 3). At RH levels below 85%, the equilibrium moisture content of DNP decreased further. When RH was 44% or lower, the equilibrium moisture content stabilized at 0.20% and showed no subsequent change. Therefore, DNP powder exhibits negligible moisture absorption at 25 °C and RH levels up to 85%.

In contrast, at RH above 85%, DNP exhibits sustained moisture absorption properties. After 240 h at 92% RH, the sample turned pale yellow with slight caking and its moisture content reached 4.82%. After 544 h, the sample partially deliquesced and liquefied. At 97% RH, the hygroscopicity of DNP was significantly enhanced. The moisture absorption rate during the first 40 h was 0.15% per hour. After the moisture content reached 6.5%, the absorption rate decreased to 0.042% per hour. By 240 h, the sample had partially deliquesced and liquefied, and by 544 h, it had completely liquefied into a saturated DNP solution. Therefore, at 25 °C, DNP continuously absorbs moisture until it completely liquefies into a saturated DNP solution when the RH exceeds 92%.

**Figure 4:**
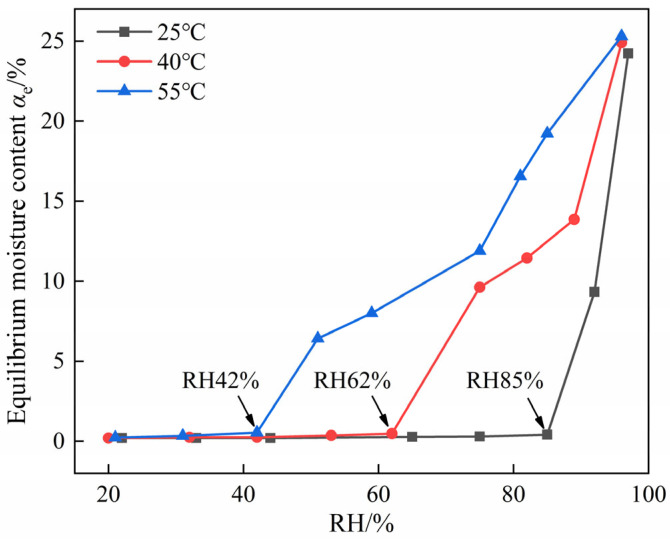
Equilibrium moisture content of DNP under varying temperature and RH conditions.

Figure 4 shows the equilibrium moisture content of DNP powder under varying temperature and RH conditions. The results determined that the CRH of DNP is 85% at 25 °C, 62% at 40 °C, and 42% at 55 °C. Therefore, the CRH of DNP decreases with increasing temperature. During the production, transportation, and storage of DNP, the RH should be controlled below the CRH corresponding to the current temperature.

**Figure 5:**
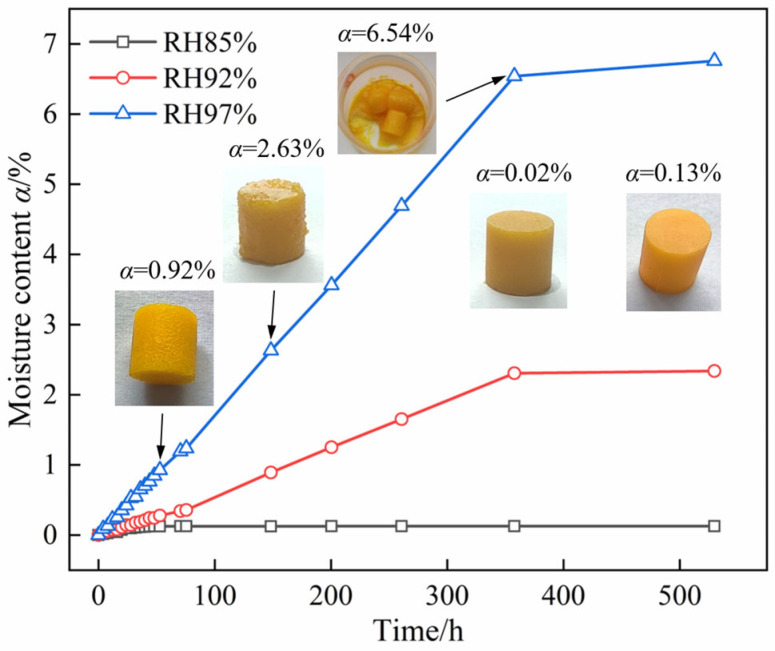
Moisture absorption curves of DNP charge at different RH values and 25 °C.

To investigate the hygroscopic properties of the DNP-based melt-cast explosive charge, we prepared cylindrical charges (10 mm in diameter and height) with a mass ratio of DNP 34.7%, Octogen (HMX) 65%, and N-Methyl-4-nitroaniline (MNA) 0.5%. Figure 5 shows the moisture absorption curves of the charge at 25 °C under varying RH conditions. At 85% RH, the equilibrium moisture content of the charge was only 0.13%, with no observable change in its appearance. The compressive strength, measured according to the GJB 772A-97 compression method, was equivalent to that of the dry charge (moisture content: 0.02%). Therefore, the CRH determined from the DNP powder was applicable to the charge. At 25 °C, the charge exhibits negligible moisture absorption when the RH remains below 85%.

At 97% RH, moisture absorption of the charge proceeds from the exterior to the interior. When the moisture content reached 0.92%, wrinkles appeared on the surface due to the moisture absorption of DNP. At a moisture content of 2.63%, partial deliquescence was observed on the surface of the charge, leading to the debonding and exposure of HMX particles from the matrix. When the moisture content reached 6.54%, the charge surface deliquesced into a supersaturated solution, resulting in a markedly reduced absorption rate, which aligns with the results of DNP powder in Figure 2.

### 2.2. Morphological Evolution of DNP upon Moisture Absorption

**Figure 6:**
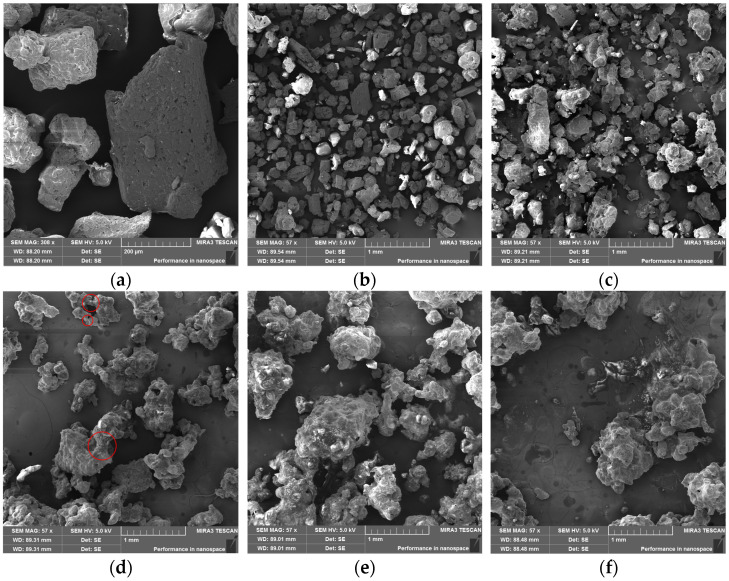
Morphology of DNP at different moisture contents at 25 °C. (**a**) dry DNP, magnified 308 times; (**b**) dry DNP, magnified 57 times; (**c**–**f**) moisture content of 4.0%, 8.0%, 6.5%, and 10.0%, respectively, magnified 57 times.

The morphology of DNP with varying moisture contents is shown in Figure 6. It must be noted that during scanning electron microscope (SEM) testing, the vacuum drying effect can remove the water from the sample, potentially altering the sample’s actual moisture content. Potential changes in the sample’s moisture content before and after SEM testing should be determined through further research. Figure 6a reveals a highly porous structure on the surface of the dry DNP particle. This structure not only significantly increases the specific surface area, providing abundant adsorption sites for water molecules in the environment, but also enables DNP to adsorb moisture through capillary condensation at lower RH.

An analysis of Figure 6b–f indicates that DNP exhibited agglomeration at a moisture content of 4.0%. When the moisture content reached 6.5%, the originally distinct interfaces and angular particles disappeared, and typical bridge structures became visible between DNP agglomerates. These observations confirm that DNP had undergone deliquescence, forming a supersaturated solution layer on the particle surface. Driven by liquid surface tension and Laplace pressure, adjacent agglomerates formed liquid bridges at their contact zones (as shown in the red circle in Figure 6d). As dissolution–recrystallization dynamic equilibrium continuously occurred within the supersaturated solution layer, the agglomerate size progressively increased. When moisture content further rose, a continuous supersaturated solution layer was observed covering the agglomerates. At this stage, moisture absorption was driven by the chemical potential gradient of water between the ambient atmosphere and the supersaturated solution layer. This proposed mechanism corroborates the results in Figure 2 and Figure 5, where the moisture absorption rate decreased when the moisture content exceeded 6.5% at 97% RH.

### 2.3. The Interaction Process Between DNP and Water Molecules

**Figure 7:**
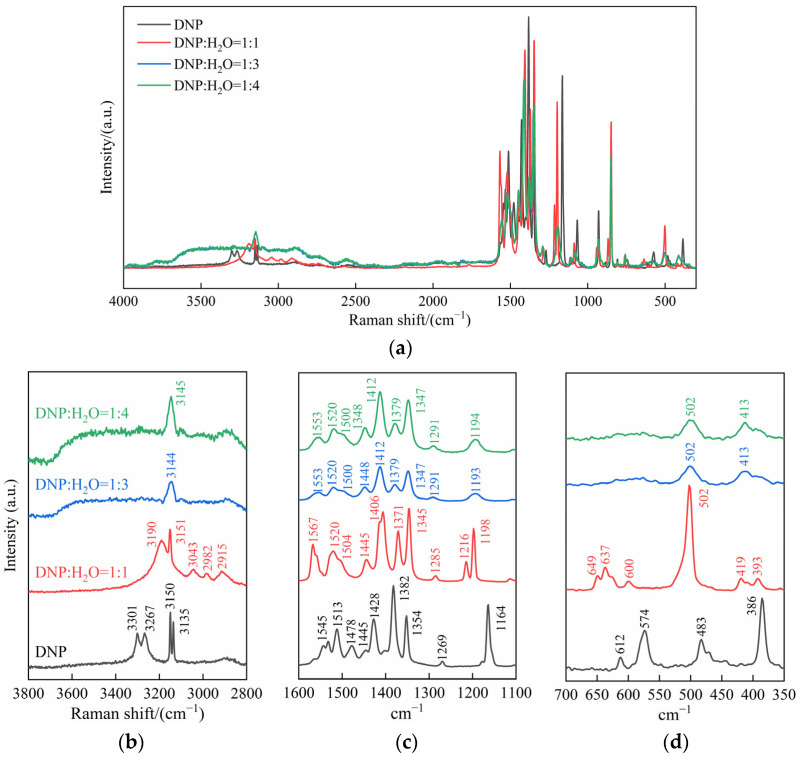
(**a**) Raman spectra of different DNP-to-water molar ratios; (**b**–**d**) partial enlargement of drawing (**a**).

Owing to the weak Raman scattering signal of water molecules and the absence of special sample preparation requirements, Raman spectroscopy proves an ideal tool for investigating subtle molecular structural changes in DNP induced by moisture absorption. Sample 1 consisted of dry DNP, while Samples 2 to 4 were prepared with DNP-to-water molar ratios of 1:1, 1:3, and 1:4, respectively (all ratios hereafter refer to molar ratios). The corresponding spectra are presented in Figure 7.

The X-ray Diffraction (XRD) of DNP confirms the presence of intermolecular hydrogen bonds within the crystal between the N-H bonds and the O or N atoms of adjacent DNP molecules [23]. The N-H bond in a DNP molecule serves as a hydrogen bond donor, forming two distinct hydrogen bonds with two adjacent DNP molecules: an N-H···O=N hydrogen bond and an N-H···N hydrogen bond. Furthermore, strong π-π stacking interactions between DNP molecules contribute to the crystal’s enhanced stability and safety. Table 1 lists the peak assignments for the 2800–3800 cm^−1^ range in Figure 7b. Compared to Sample 1, the N-H stretching vibration peak in Sample 2 exhibited a significant redshift and peak broadening. This suggests that water molecules, acting as stronger hydrogen bond acceptors, partially replaced the original N-H···O/N hydrogen-bond network in the DNP crystal. At a DNP-to-water ratio of 1:3 (Sample 3), a broad O-H stretching peak appeared in the 2700–3800 cm^−1^ range, confirming the presence of abundant unbound water in Sample 3, indicating the formation of a solution with DNP as the solute. Concurrently, the N-H stretching vibration peak underwent a further redshift and merged into a broad peak at 3144 cm^−1^, indicating that the N-H bonds of DNP molecules formed hydrogen bonds with water molecules in the aqueous solution with nearly uniform strength. Further increasing the water ratio yielded no changes in the Raman spectra, confirming that the system had reached a fully hydrated state.

Peak assignments for the 1100–1600 cm^−1^ and 350–700 cm^−1^ ranges in Figure 7c,d are summarized in Table 2 and Table 3, respectively. With increasing water ratio, the nitro asymmetric stretching vibration peak in Sample 2 exhibited a significant blueshift from 1545 cm^−1^ to 1567 cm^−1^. This is attributed to water molecules acting as hydrogen bond donors to form O-H···O=N bonds with the nitro group, which increases the N=O bond force constant. Conversely, the nitro symmetric stretching vibration peak redshifted from 1382 cm^−1^ to 1371 cm^−1^. This shift may result from the hydrogen bonds weakening the intramolecular conjugation effect between the nitro group and the pyrazole ring. This phenomenon demonstrates the distinct effects of hydrogen bonds on different vibrational modes of nitro group.

Samples 3 and 4 have reached a fully hydrated state. Here, the water molecules hydrogen-bonded to the nitro groups further engage with other water molecules to form a dynamically stable hydrogen-bond network. In contrast, when a nitro group forms a hydrogen bond with a single water molecule in Sample 2, it more readily adopts a linear O-H···O three-atom configuration. This configuration leads to the strongest hydrogen bond between the nitro group and the water molecule, resulting in the largest observed peak shift for the nitro group. Consequently, compared to Sample 2, the peak shift in the nitro group is slightly reduced in Samples 3 and 4. Conversely, the peak shift in the N-H bond increased. This is attributed to the limited number of water molecules in Sample 2, which prevents all N-H bonds from forming hydrogen bonds with water molecules. In a solution environment (Samples 3 and 4), however, the N-H bonds can fully hydrate, participating in the formation of a stable hydrogen-bond network.

As indicated in Table 2 and Table 3, compared to Sample 1, the peaks associated with the entire pyrazole ring and its skeleton (such as C=N, C=C, C-N, and C-C bonds) in Sample 2 generally exhibited blueshifts. This demonstrates that water molecules compete with and disrupt the original π-π stacking interactions and intermolecular hydrogen-bond networks within the DNP crystal, leading to a relative increase in the electron density of the pyrazole ring and an enhanced bond order. Following the formation of DNP solutions (Samples 3 and 4), the peak shifts in the nitro group and pyrazole ring skeleton slightly decreased and stabilized. This indicates the establishment of a dynamically stable hydrogen-bond network between DNP and water molecules.

Through comprehensive analysis of Raman spectra, the moisture absorption mechanism of DNP can be elucidated at the molecular level. Water molecules, acting as both effective hydrogen bond acceptors and donors, form hydrogen bonds with the N-H bonds and nitro groups in DNP molecules, respectively. This configuration is consistent with the most stable structure identified through natural bond orbital (NBO) analysis in Reference [25]. The hydrogen bonds formed by water molecules compete with and subsequently replace the original N-H···O/N hydrogen bond network within DNP crystals, disrupting the intermolecular π-π stacking interactions. This is confirmed by the general blueshift in the peaks of the entire pyrazole ring and its skeleton. When the DNP-to-water molar ratio exceeds 1:3, the wavenumbers of all absorption peaks stabilize, indicating the establishment of a dynamically stable hydrogen-bond network between DNP and water molecules.

## 3. Materials and Methods

### 3.1. Materials

DNP, 99.5% purity; Octogen (HMX); N-Methyl-4-nitroaniline (MNA), 99% purity; Chongqing Hongyu Precision Industry Co. Ltd. (Chongqing, China). Sodium bromide (NaBr), AR; Potassium chloride (KCl), GR; Potassium sulfate (K_2_SO_4_), AR; Potassium acetate (CH_3_COOK), AR; Magnesium chloride (MgCl_2_), CP; Potassium carbonate (K_2_CO_3_), AR; Sodium nitrite (NaNO_2_), AR; Sodium chloride (NaCl), GR; Sinopharm chemical reagent Co., Ltd. (Shanghai, China).

### 3.2. Methods

#### 3.2.1. Initial Moisture Content

The initial moisture content of samples was determined using the Mettler Toledo V30S Karl Fischer moisture analyzer (Mettler-Toledo (China), Co., Ltd., Shanghai, China). Each sample weighed 1.0 ± 0.02 g. All measurements were performed in two replicates, and the data are reported as mean values.

#### 3.2.2. Moisture Content

DNP powder was first dried in a constant-temperature oven at 55 °C for 4 h. Its moisture content was then determined following Section 3.2.1 to confirm an initial value of less than 0.02%. A supersaturated salt solution was prepared in the desiccator to establish a specific RH environment according to the GJB770B-2005 hygroscopicity-dryer balance method [26]. After the temperature and RH inside the desiccator stabilized, 2.0 ± 0.02 g of the dried powder or three DNP/HMX/MNA charges were placed into a Petri dish. The dish was then placed inside the desiccator and the entire desiccator was placed into the constant-temperature oven. The Petri dish was removed and weighed at 4 h intervals using a BSA224S-CW analytical balance (Nanjing Laibu Technology Industrial Co., Ltd., Hanjing, China). The moisture content was calculated as follows:

$$\alpha =\frac{M-M_{0}}{M}\times 100\%$$

where α—moisture content; $M_{0}$—the weight of sample before absorbing moisture; M—the weight of the sample after absorbing moisture.

#### 3.2.3. Scanning Electron Microscope (SEM)

The sample with known moisture content was thinly dispersed on the sample stage and sputter-coated with a gold layer. Then the sample was imaged using a TESCAN MIRA3 XMU field emission scanning electron microscope (TESCAN Trading (Shanghai) Co., Ltd., Shanghai, China).

#### 3.2.4. Raman Spectroscopy

Raman spectra were acquired using a LabRAM HR Evolution micro-confocal Raman spectrometer (HORIBA (China) Trading Co., Ltd., Shanghai, China). We employed a 532 nm laser at its 50% power level (50 mW) to perform the measurements. To verify whether the thermal effect of the laser influenced the test results, the same sampling point was subjected to three consecutive and repeated scans. The completely superimposed Raman spectra confirmed that the laser thermal effect was negligible. To prevent fortuitous results, three distinct sampling points were scanned for each sample. The data were considered valid only when all collected spectra were consistent. The baseline was automatically corrected using the LabSpec 5 software.

## 4. Conclusions

This work focused on elucidating the hygroscopic properties and mechanisms of the novel explosive DNP, with the purpose of providing a theoretical basis for solving its hygroscopicity. We measured the moisture absorption curves of DNP powder and DNP/HMX/MNA charges under varying temperature and RH conditions, observed the morphological evolution of DNP at different moisture contents, and analyzed the Raman spectra of samples with different molar ratios of DNP to water. The conclusions are as follows. The hygroscopicity of DNP intensifies with rising temperature and RH. The CRH was determined to be 85% at 25 °C, 62% at 40 °C, and 42% at 55 °C. DNP exhibits negligible moisture absorption when the RH is below CRH. At the micrometer scale, the highly porous structure on the surface of DNP particles facilitates moisture absorption. Upon absorbing moisture, the particle surface first transforms into a supersaturated DNP solution layer. Driven by liquid surface tension and Laplace pressure, this solution then forms liquid bridges between adjacent particles at their contact zones. The continuous dissolution–recrystallization dynamic process within the supersaturated solution layer promotes agglomeration, ultimately leading to deliquescence into a saturated solution. At the molecular scale, water molecules form hydrogen bonds with both the N-H bonds and nitro groups of DNP molecules. This process supersedes the original hydrogen-bond network within DNP crystals and disrupts the π-π stacking interactions. When the molar ratio of DNP to water exceeds 1:3, the Raman spectra remain unchanged, indicating the attainment of a fully hydrated state.

## Figures and Tables

**Table 1:** Peak assignments in the 2800–3800 cm^−1^ wavenumber range.

Vibration Mode	Wavenumber/cm^−1^			
	Sample 1	Sample 2	Sample 3	Sample 4
N-H stretching	3301, 3267	3190, 3151	3144	3144
C-H stretching	3150, 3135	3043, 2982	-	-
O-H stretching (H_2_O)	-	2915	2800–3700	2800–3700

**Table 2:** Peak assignments in the 1100–1600 cm^−1^ wavenumber range.

Vibration Mode	Wavenumber/cm^−1^			
	Sample 1	Sample 2	Sample 3	Sample 4
-NO_2_ asymmetric stretching	1545	1567	1553	1553
C=N stretching	1513	1520	1520	1520
C=C stretching	1478	1504	1500	1500
N-H in-plane bending	1445	1445	1448	1448
C-H in-plane bending	1428	1406	1412	1412
-NO_2_ symmetric stretching	1382, 1354	1371, 1345	1379, 1347	1379, 1347
C-N stretching	1269	1285	1291	1291
C-C stretching	1164	1216, 1198	1193	1194

**Table 3:** Peak assignments in the 350–700 cm^−1^ wavenumber range.

Vibration Mode	Wavenumber/cm^−1^			
	Sample 1	Sample 2	Sample 3	Sample 4
pyrazole ring global out-of-plane deformation	612	649, 637	-	-
C=C out-of-plane bending	574	600	-	-
N-H out-of-plane bending	483	502	502	502
pyrazole ring out-of-plane bending	386	419, 393	413	413

## Data Availability

The data in this work are available from the authors.
